# Supplementary material for: The Stress-Responsive microRNA-34a Alters Insulin Signaling and Actions in Adipocytes through Induction of the Tyrosine Phosphatase PTP1B
Source: Cells. 2022 Aug 19;11(16):2581. doi: 10.3390/cells11162581 (PMC9406349; doi:10.3390/cells11162581)
Supplement: Supplementary file 1 [file cells-11-02581-s001.zip › cells-1798961-supplementary.pdf]

cells

MDPI

Supplementary Materials

The Stress-Responsive microRNA-34a Alters Insulin Signaling and Actions in Adipocytes through Induction of the Tyrosine Phosphatase PTP1B

Pierre-Jean Cornejo [1], Bastien Vergoni [1], Mickaël Ohanna [2], Brice Angot [1], Teresa Gonzalez [1,3], Jennifer Jager [1], Jean-François Tanti [1] and Mireille Cormont [1,*]

1 Université Côte d'Azur, Inserm, C3M, Team "Molecular and Cellular Physiopathology of Obesity and Diabetes", 06204 Nice, France
2 Université Côte d'Azur, Inserm, C3M, Team "Microenvironnement, Signalisation et Cancer", 06204 Nice, France
3 Aix Marseille Université, INSERM, INRAE, C2VN, 13385 Marseille, France
* Correspondence: mireille.cormont@univ-cotedazur.fr; Tel.: +33-4-89-15-38-31

Citation: Cornejo, P.-J.; Vergoni, B.; Ohanna, M.; Angot, B.; Gonzalez, T.; Jager, J.; Tanti, J.-F.; Cormont, M. The Stress-Responsive microRNA-34a Alters Insulin Signaling and Actions in Adipocytes through Induction of the Tyrosine Phosphatase PTP1B. *Cells* **2022**, *11*, 2581. https://doi.org/10.3390/cells11162581

Academic Editors: Say Viengchareun, Maria Rosa Ciriolo and Fabio Ciccarone

Received: 17 June 2022
Accepted: 5 August 2022
Published: 19 August 2022

Publisher's Note: MDPI stays neutral with regard to jurisdictional claims in published maps and institutional affiliations.

Copyright: © 2022 by the authors. Licensee MDPI, Basel, Switzerland. This article is an open access article distributed under the terms and conditions of the Creative Commons Attribution (CC BY) license (https://creativecommons.org/licenses/by/4.0/).

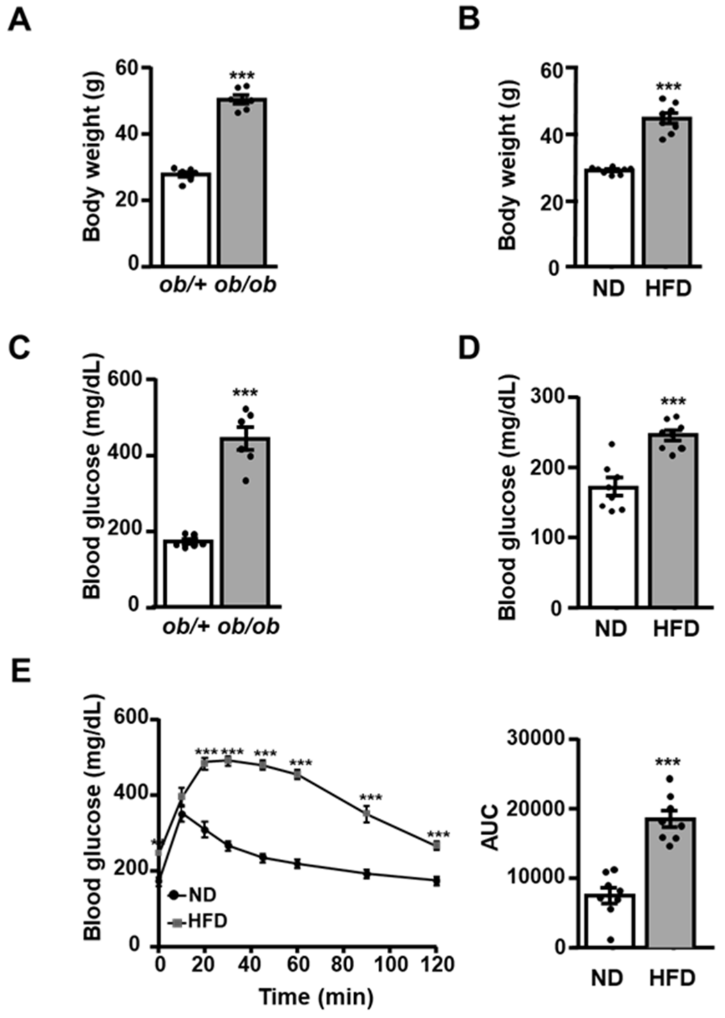

Figure S1. (**A**) Body weight of the genetically obese *ob*/*ob* mice and their lean *ob*/+ littermates (n = 6). (**B**) Body weight of wild-type (WT) chow-fed mice (ND) and HFD-fed mice (n = 8). (**C**) Fed glycemia of the genetically obese *ob*/*ob* mice and their lean *ob*/+ lit-

termates (n = 6). (**D**) Glycemia of WT chow-fed mice (ND) and HFD-fed mice (n = 8). (**E**) Intraperitoneal glucose tolerance test (IPGTT) of WT chow-fed and HFD-fed mice with area under the curve (AUC) quantitated in the bar graph (n = 8). Bar graphs are mean values ± SEM. *** $p < 0.001$. Unpaired two-tailed Student t-test was used for the comparison of two conditions in the bar graphs. Two-way ANOVA followed by Bonferonni's posttest was used for the comparison of the IPGTT curve.

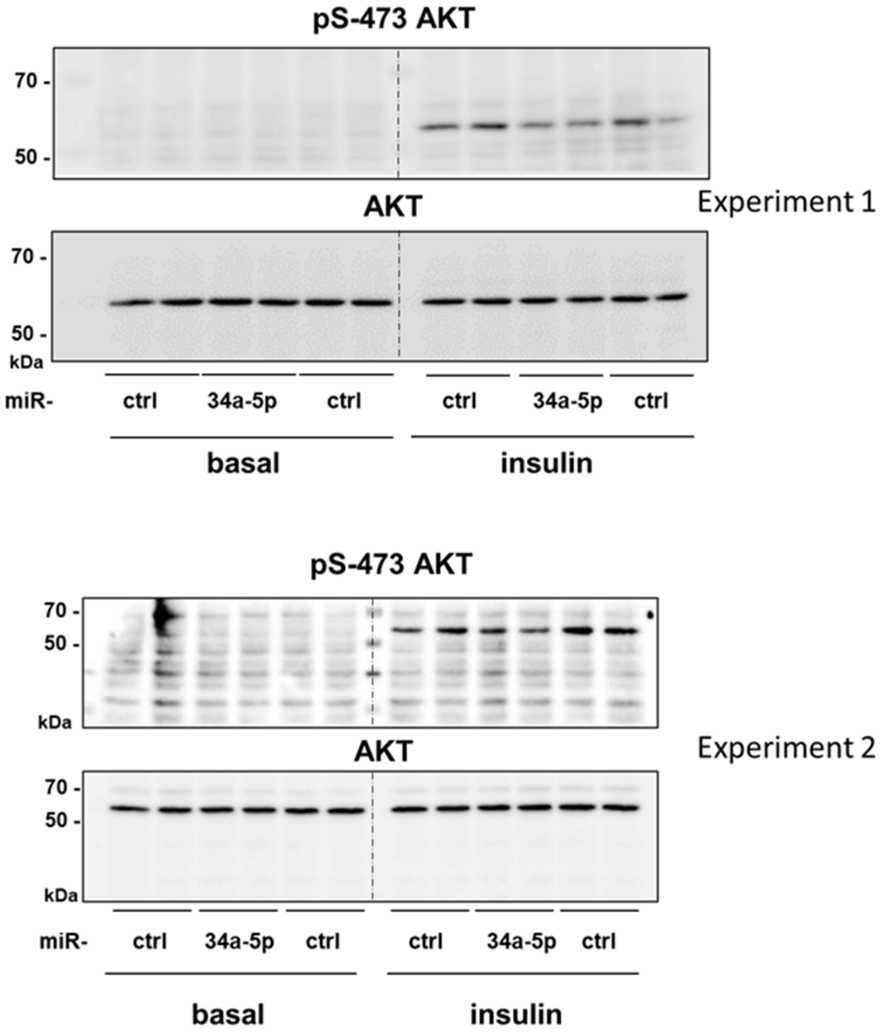

Figure S2. Immunoblot from two independent experiments of the level of phosphoSer473-AKT (pS-473 AKT) and total AKT in 3T3-L1 adipocytes transfected with a control miRNA (miR-Ctrl) or a miR-34a mimic (miR-34a-5p) and treated without or with insulin (0.5 nM) for 10 min.
